# Correction: Phan et al. Injectable Hydrogel Based on Protein-Polyester Microporous Network as an Implantable Niche for Active Cell Recruitment. *Pharmaceutics* 2022, *14*, 709

**DOI:** 10.3390/pharmaceutics18101312

**Published:** 2026-10-16

**Authors:** V.H. Giang Phan, Mohanapriya Murugesan, Panchanathan Manivasagan, Thanh Loc Nguyen, Thuy-Hien Phan, Cuong Hung Luu, Duy-Khiet Ho, Yi Li, Jaeyun Kim, Doo Sung Lee, Thavasyappan Thambi

**Affiliations:** 1 Biomaterials and Nanotechnology Research Group, Faculty of Applied Sciences, Ton Duc Thang University, Ho Chi Minh City 70000, Vietnam; 2 Graduate School of Biotechnology, College of Life Sciences, Kyung Hee University, Yongin-si 17104, Gyeonggi-do, Republic of Korea; 3 Department of Applied Chemistry, Kumoh National Institute of Technology, Daehak-ro 61, Gumi-si 39177, Gyeongbuk, Republic of Korea; 4 School of Chemical Engineering, Sungkyunkwan University, Suwon 16419, Gyeonggi-do, Republic of Korea; 5 Department of Bioengineering, University of Washington, Seattle, WA 98195, USA; 6 College of Materials and Textile Engineering, Nanotechnology Research Institute, Jiaxing University, Jiaxing 314001, China

## Correction to References

In the original publication [1], References 20–24 were added during the peer-review process following a reviewer’s recommendation. Although these references were broadly related to hydrogels and polymeric drug-delivery systems, the authors have reassessed their relevance to the corresponding statements. To improve the accuracy and objectivity of the reference list, References 20–24 have been replaced with more appropriate references [2,3,4,5,6], which better support the corresponding scientific statements.

“Hydrogels prepared using natural polymers such as chitosan, zein and gelatin by cross-linking exhibited good stimuli-responsive characteristics and controlled release characteristics [2,3,4,5]. In addition, poly(caprolactone)-based synthetic hydrogels showed sharp sol-to-gel phase transition and prolonged the release of therapeutics [6].”

The authors state that the scientific conclusions are unaffected. This correction was approved by the Academic Editor. The original publication has also been updated.
